# Compression for Bayer CFA Images: Review and Performance Comparison

**DOI:** 10.3390/s22218362

**Published:** 2022-10-31

**Authors:** Kuo-Liang Chung, Hsuan-Ying Chen, Tsung-Lun Hsieh, Yen-Bo Chen

**Affiliations:** Department of Computer Science and Information Engineering, National Taiwan University of Science and Technology, Taipei 10672, Taiwan

**Keywords:** Bayer color filter array (CFA) images, compression-first-based compression, chroma subsampling-then-luma modification, demosaicing-first-based compression, joint photographic experts group-2000 (JPEG-2000), quality–bitrate tradeoff, reversible color transform-based compression, Versatile Video Coding (VVC)

## Abstract

Bayer color filter array (CFA) images are captured by a single-chip image sensor covered with a Bayer CFA pattern which has been widely used in modern digital cameras. In the past two decades, many compression methods have been proposed to compress Bayer CFA images. These compression methods can be roughly divided into the compression-first-based (CF-based) scheme and the demosaicing-first-based (DF-based) scheme. However, in the literature, no review article for the two compression schemes and their compression performance is reported. In this article, the related CF-based and DF-based compression works are reviewed first. Then, the testing Bayer CFA images created from the Kodak, IMAX, screen content images, videos, and classical image datasets are compressed on the Joint Photographic Experts Group-2000 (JPEG-2000) and the newly released Versatile Video Coding (VVC) platform VTM-16.2. In terms of the commonly used objective quality, perceptual quality metrics, the perceptual effect, and the quality–bitrate tradeoff metric, the compression performance comparison of the CF-based compression methods, in particular the reversible color transform-based compression methods and the DF-based compression methods, is reported and discussed.

## 1. Introduction

**Figure 1:**
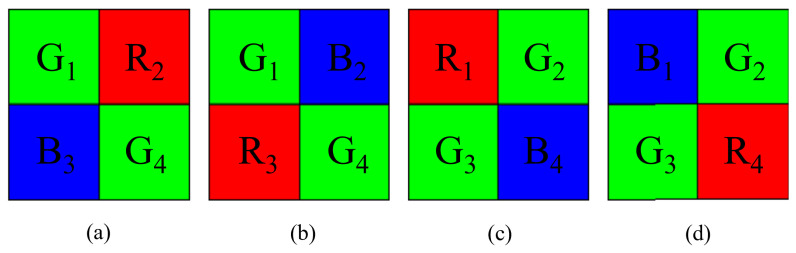
Four 2 × 2 Bayer CFA patterns. (**a**) $Pat_{1}$ = [$G_{1}$, $R_{2}$, $B_{3}$, $G_{4}$]. (**b**) $Pat_{2}$ = [$G_{1}$, $B_{2}$, $R_{3}$, $G_{4}$]. (**c**) $Pat_{3}$ = [$R_{1}$, $G_{2}$, $G_{3}$, $B_{4}$]. (**d**) $Pat_{4}$ = [$B_{1}$, $G_{2}$, $G_{3}$, $R_{4}$].

**Figure 2:**
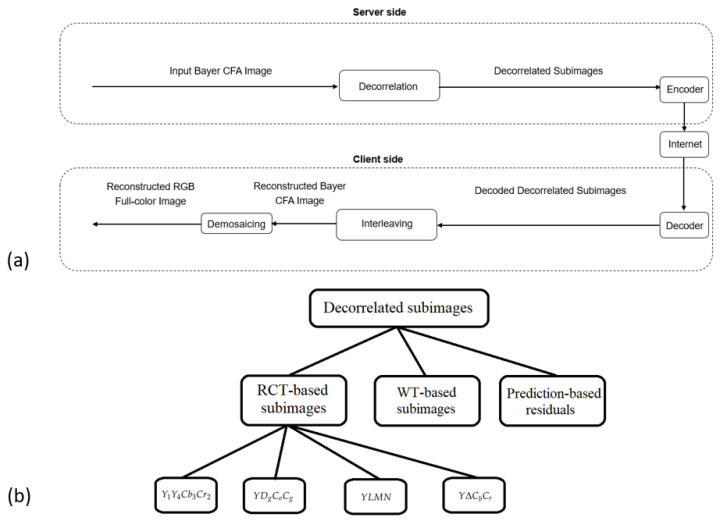
The CF-based compression scheme for $I^{Bayer}$. (**a**) The server side and the client side. (**b**) In terms of the decorrelated subimages, the graphical representation of the presented CF-based methods.

**Figure 3:**
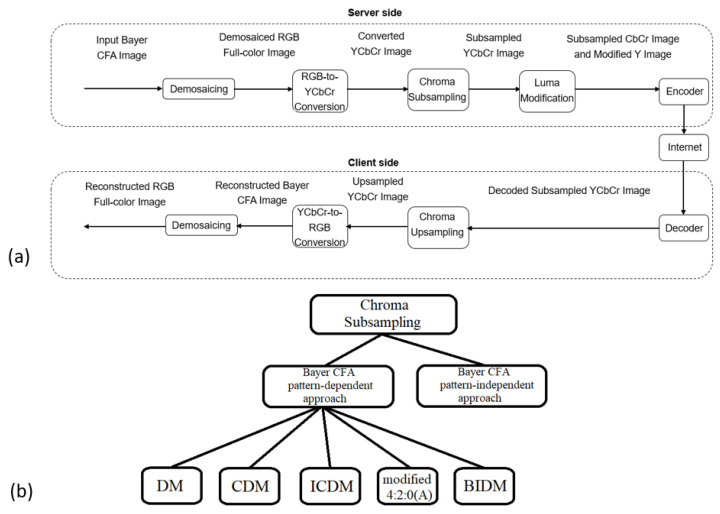
The DF-based compression scheme for $I^{Bayer}$. (**a**) The server side and the client side. (**b**) The graphical representation of chroma subsampling, particularly the Bayer CFA pattern-independent chroma subsampling approach and the Bayer CFA pattern-dependent chroma subsampling approach.

To save hardware costs, most color digital cameras employ single-sensor technologies with Bayer color filter array (CFA) patterns to capture real-world scenes. The four widely used 2 × 2 Bayer CFA patterns [1], namely $Pat_{1}$ = [$G_{1}$, $R_{2}$, $B_{3}$, $G_{4}$], $Pat_{2}$ = [$G_{1}$, $B_{2}$, $R_{3}$, $G_{4}$], $Pat_{3}$ = [$R_{1}$, $G_{2}$, $G_{3}$, $B_{4}$], and $Pat_{4}$ = [$B_{1}$, $G_{2}$, $G_{3}$, $R_{4}$], are shown in Figure 1a–d, respectively. For convenience, the captured Bayer CFA raw image is denoted by I${}^{Bayer}$, in which each pixel has only one R (red), G (green), or B (blue) color value and I${}^{Bayer}$ consists of 25% R, 50% G, and 25% B color values. To fully utilize the limited device resources of cameras, such as the limited storage space and transmission capacity, prior to storing or transmitting Bayer CFA images, compressing Bayer CFA images is necessary. Without the loss of generality, in our discussion for compressing $I^{Bayer}$, we only consider the first Bayer CFA pattern in Figure 1a, but our discussion is also applicable to the other three CFA patterns. During the past two decades, many compression methods for $I^{Bayer}$ have been developed and they can be roughly divided into two schemes, namely the compression-first-based (CF-based) scheme in Figure 2a and the demosaicing-first-based (DF-based) scheme in Figure 3a. In both compression schemes, at the client side, besides the reconstructed Bayer CFA image $I^{rec,Bayer}$, the reconstructed RGB full-color image, which is obtained by demosaicing $I^{rec,Bayer}$, is also used to evaluate the quality and quality–bitrate performance of the related compression methods. It is noticeable that the input Bayer CFA image $I^{Bayer}$ and the demosaiced RGB full-color image $I^{demo,RGB}$ at the server side of Figure 2a and Figure 3a are used as the ground-truth Bayer CFA image and the ground-truth reconstructed RGB full-color image, respectively.

### 1.1. The Related CF-Based Compression Methods

At the server side of Figure 2a, the input Bayer CFA image $I^{Bayer}$ is first decorrelated to some subimages. In Figure 2b, the decorrelated subimages could be the reversible color transform-based (RCT-based) subimages [2,3,4,5,6,7,8,9], the wavelet transform-based (WT-based) subbands [10,11,12,13,14], or the prediction-based residuals [10,15].

In the RCT-based decorrelated subimages, the four RCT-based formats, namely the $Y_{1}Cr_{2}Cb_{3}Y_{4}$ format proposed by Lee and Ortega [2] and implemented on JPEG [16], the $YD_{g}C_{o}C_{g}$ format proposed by Malvar and Sullivan [3] and implemented on JPEG-XR [17] and JPEG-2000 [18,19], the YLMN format proposed by Mohammed et al. [4] and implemented on Golomb–Rice codec [20] and JPEG-2000, and the $Y\Delta C_{b}C_{r}$ format proposed by Richter and Fößel [8] and implemented on JPEG-XS [21], have received growing attention. For the $YD_{g}C_{o}C_{g}$ representation of $I^{Bayer}$, Suzuki [12] proposed a lossless WT-based spectral–spatial transformation (WSST) approach to improve the bitrate performance. To improve WSST, Suzuki [14] proposed a weighted version by taking the edge directions into account. For improving the compression performance, Richter et al. [9] not only performed a nonlinear gamma correction on $I^{Bayer}$ but also deployed two white-balance constants into the two luma components in the Star-Tetrix transformation-based representation which was implemented on JPEG-XS [21].

Zhang and Wu [10] proposed a lossless merge- and residual-based method for compressing $I^{Bayer}$. As a result, the rectangular compact green subimage, the red residual subimage, and blue residual subimage are fed into the codec. Chung and Chan [15] proposed a lossless context matching-based prediction method, in which when predicting the current pixel, the neighboring pixels of the current pixel are ranked, to obtain more accurate residual red and blue subimages, achieving a better compression performance relative to the method [10] on the Rice encoder. Zhang and Wu [10] performed a Mallat wavelet transform [22] on $I^{Bayer}$, and then, the Golomb–Rice encoder [20] was utilized to encode the transformed wavelet coefficients. Lee et al. [11] proposed a camera-aware multi-resolution analysis (CAMRA) framework for compressing $I^{Bayer}$. They leveraged the decorrelated wavelet coefficients and the image pipeline techniques at the server side. Later, Lee and Hirakawa [13] proposed a new shift-and-decorrelate lifting method to improve the compression performance of CAMRA.

We now explain why we only consider the RCT-based compression methods in the above-mentioned CF-based compression methods and select JPEG-2000 as the compression platform. In the literature, the RCT-based compression methods often served as the main comparative methods. Among these codecs used to evaluate the compression performance of the considered compression methods, JPEG-2000 is most favored. In addition, the prediction-based residual approaches [10,15] are lossless; the WT-based approaches [10,11,12,13,14] involve different codecs, such as the Golomb–Rice encoder and JPEG-2000, various WT-based computations, such as Haar, 5/3, and 9/7 wavelet transforms, varying matrix chain multiplications, and lifting operations. Accordingly, we take the RCT-based methods as the representatives of the CF-based methods. Section 2 will introduce the above-mentioned four RCT-based methods in detail. Furthermore, JPEG-2000 is adopted to evaluate the compression performance of the considered compression methods.

### 1.2. The Related DF-Based Compression Methods

In Figure 3a, at the server side of the DF-based compression scheme, the input Bayer CFA image $I^{Bayer}$ is first demosaiced to an RGB full-color image $I^{demo,RGB}$ which also serves as the ground-truth RGB full-color image. Section 3.1.1 will introduce how to demosaic $I^{Bayer}$ to $I^{demo,RGB}$. Next, $I^{demo,RGB}$ is converted to a YCbCr image $I^{YCbCr}$. Section 3.1.2 will introduce an RGB-to-YCbCr transformation. Then, a chroma 4:2:0 subsampling method is performed on the chroma image $I^{CbCr}$ to obtain a subsampled CbCr image $I^{sub,CbCr}$ whose size is a quarter of the original chroma image $I^{CbCr}$. In Section 3.2, two kinds of chroma 4:2:0 subsampling approaches [23,24,25,26,27,28,29,30,31,32], namely the Bayer CFA pattern-independent approach and the Bayer CFA pattern-dependent approach in Figure 3b, will be introduced. Furthermore, based on the subsampled CbCr image $I^{sub,CbCr}$ and the luma image $I^{Y}$, a luma modification method is performed on $I^{Y}$ to obtain a modified luma image $I^{mod,Y}$. Section 3.3 will introduce the two related luma modification methods [33,34].

As a result, the subsampled CbCr image $I^{sub,CbCr}$ and the modified luma image $I^{mod,Y}$ are fed into the encoder. At the client side, a chroma upsampling method is first performed on the decompressed subsampled CbCr image to construct the upsampled CbCr image. Finally, a YCbCr-to-RGB transformation is performed on the upsampled YCbCr image to obtain the reconstructed Bayer CFA image $I^{rec,Bayer}$, and then as mentioned before, $I^{rec,Bayer}$ is demosaiced to an RGB full-color image which serves as the reconstructed RGB full-color image. Among the codecs used to evaluate the compression performance of the considered DF-based compression methods, the Versatile Video Coding (VVC) platform [35] is most favored.

### 1.3. Motivation and Contribution

To date, in the literature, no review article for the above-mentioned CF-based compression scheme and the DF-based compression scheme for Bayer CFA images has been published. Besides that, no compression performance comparison of the two compression schemes has been reported. Therefore, it motivated us to review the related CF-based compression works, in particular the related RCT-based compression works and the DF-based works. In addition, it motivated us to compare and discuss the compression performance for the considered compression methods on JPEG-2000 and the newly released VVC platform VTM-16.2.

In the RCT-based compression methods, the four main methods, namely the $Y_{1}Y_{4}Cb_{3}Cr_{2}$ method [2], the $YD_{g}C_{o}C_{g}$ method [3], the YLMN method [4], and the $Y\Delta C_{b}C_{r}$ method [8], are introduced in detail. To evaluate the compression performance of these compression methods on JPEG-2000 and VTM-16.2, considering simplicity and effectiveness, the three methods, namely the $YD_{g}C_{o}C_{g}$ method [3], the YLMN method [4], and the $Y\Delta C_{b}C_{r}$ method [8], are selected as the representatives.

In the DF-based compression scheme, besides the demosaicing part, the other macro-part is called the “chroma subsampling-then-luma modification (CSLM)” macro-part which consists of the chroma subsampling part and the luma modification part. In the chroma subsampling part, we first introduce eight Bayer CFA pattern-independent chroma subsampling methods: 4:2:0(A), 4:2:0(L), 4:2:0(R), 4:2:0(DIRECT), 4:2:0(MPEG-B) [23], the Anchor method [24], the interpolation-dependent image downsampling (IDID) method [25], and the joint chroma downsampling and upsampling (JCDU) method [26]. In Figure 3b, we further introduce five Bayer CFA pattern-dependent chroma subsampling methods: the direct mapping (DM) method [27], the COPY-based distortion minimization (CDM) method [28], the improved CDM (ICDM) method [29], the modified 4:2:0(A) method [30], and the bilinear interpolation-based distortion minimization (BIDM) method [31]. In the luma modification part, we introduce two luma modification methods: the Bayer CFA pattern-independent LM method [33] and the optimal Bayer CFA pattern-dependent LM (OLM) method [34]. Considering the effectiveness, the three CSLM methods, namely CDM-OLM, “modified 4:2:0(A)”-OLM, and BIDM-OLM, are selected as the representatives to evaluate the compression performance of the DF-based compression scheme.

Based on the testing Bayer CFA images created from the Kodak, IMAX, screen content images (SCI), Videos, and classical images (CI) datasets, thorough experiments have been carried out for the above-mentioned representatives of the CF-based and DF-based compression schemes on JPEG-2000 and VTM-16.2. When setting the quantization parameter (QP) to 0 for VTM-16.2 and setting the compression ratio (CR) to 1 for JPEG-2000, in terms of the three popular quality metrics, namely the peak signal-to-noise ratio (PSNR), the structure similarity (SSIM) [36], and the feature similarity (FSIM) [37], the $YD_{g}C_{o}C_{g}$ method achieves the best PSNR performance, and the BIDM-OLM method is ranked second; the BIDM-OLM method achieves the best SSIM and FSIM performance, and the $YD_{g}C_{o}C_{g}$ method is ranked second. On JPEG-2000, in terms of the widely used quality–bitrate tradeoff metric, namely the Bjøntegaard delta (BD)-PSNR [38], the BIDM-OLM method always achieves the best BD-PSNR performance. On VTM-16.2, the $YD_{g}C_{o}C_{g}$ method achieves the best BD-PSNR performance under the high bitrate circumstance, while under the middle and low bitrate circumstances, the BIDM-OLM method achieves the best BD-PSNR performance. In addition, the perceptual quality comparison and the execution time requirement comparison are also made. Finally, some future works are addressed.

The remainder of this article is organized as follows. In Section 2, the related RCT-based compression works for $I^{Bayer}$ are introduced. In Section 3, the related DF-based compression works for $I^{Bayer}$ are introduced. In Section 4, the compression performance comparison between the two compression schemes are provided. In Section 5, some concluding remarks and future works are addressed.

## 2. The Reversible Color Transform-Based (RCT-Based) Compression Works for Bayer CFA Images

**Figure 4:**
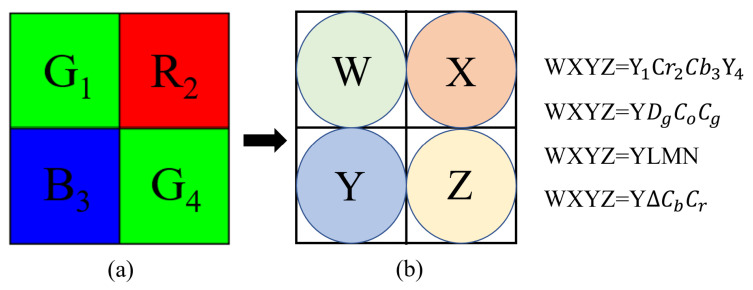
The relation between the 2 × 2 Bayer CFA block and the four RCT-based formats. (**a**) The 2 × 2 Bayer CFA block. (**b**) The four RCT-based formats.

For $I^{Bayer}$, we mainly introduce the four RCT-based compression methods: the $YD_{g}C_{o}C_{g}$ method [3], the YLMN method [4], and the $Y\Delta C_{b}C_{r}$ method [8]. Figure 4 depicts the relation between the 2 × 2 Bayer CFA block and the four RCT-based formats.

### 2.1. The $Y_{1}Cr_{2}Cb_{3}Y_{4}$ Method

Lee and Ortega [2] proposed a $Y_{1}Cr_{2}Cb_{3}Y_{4}$ method to decorrelate the input Bayer CFA image $I^{Bayer}$ to four subimages, namely the $Y_{1}$, $Cr_{2}$, $Cb_{3}$, and $Y_{4}$ subimages such that $I^{Y_{1}Cr_{2}Cb_{3}Y_{4}}$ can be better compressed by JPEG. First, each 2 × 2 Bayer CFA block $B^{Bayer}$ is converted to a 2 × 2 $Y_{1}Cr_{2}Cb_{3}Y_{4}$ block in Figure 4b by using the following formula:

(1) $$\left[\begin{array}{c} Y_{1}\\ Y_{4}\\ Cb_{3}\\ Cr_{2} \end{array}\right]=\left[\begin{array}{cccc} 0.587 & 0 & 0.114 & 0.299\\ 0 & 0.587 & 0.114 & 0.299\\ -0.170 & -0.170 & 0.511 & -0.172\\ -0.214 & -0.214 & -0.083 & 0.511 \end{array}\right]\left[\begin{array}{c} G_{1}\\ G_{4}\\ B_{3}\\ R_{2} \end{array}\right]+\left[\begin{array}{c} 0\\ 0\\ 128\\ 128 \end{array}\right]$$

where $Y_{1}$ and $Y_{4}$ denote the two converted luma components, and $Cb_{3}$ and $Cb_{2}$ denote the converted chroma components.

**Figure 5:**
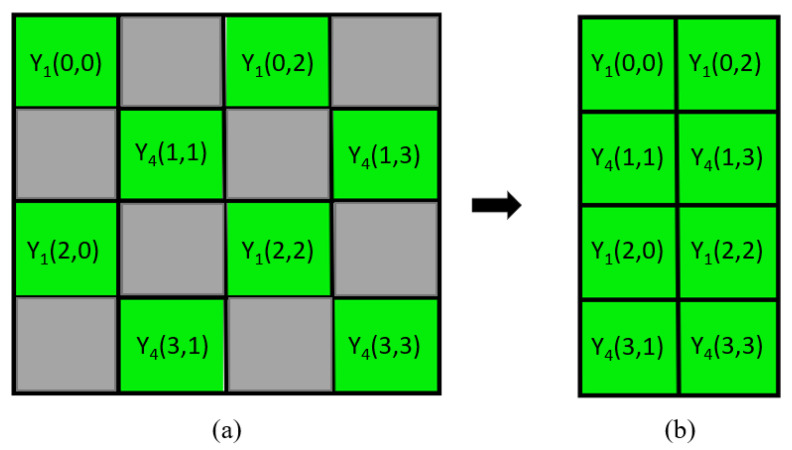
The construction of the rectangular compact luma image. (**a**) The quincunx-located luma image. (**b**) The rectangular compact luma image.

On the other hand, by Equation (1), one input Bayer CFA image $I^{Bayer}$ is converted into a $Y_{1}Cr_{2}Cb_{3}Y_{4}$ image $I^{Y_{1}Cr_{2}Cb_{3}Y_{4}}$ which contains four subimages, namely the two luma subimages, $I^{Y_{1}}$ and $I^{Y_{4}}$, constituting a quincunx-located luma image in Figure 5a, and the two chroma subimages, $I^{Cb_{3}}$ and $I^{Cr_{2}}$. In order to produce a compact luma image containing $I^{Y_{1}}$ and $I^{Y_{4}}$, every luma value in each even column of the quincunx-located image is shifted left to the odd column, and then, each even column is removed. Figure 5b shows the rectangular compact luma image. Next, the rectangular compact luma image is rotated 45 degree clockwise to produce a rhombic compact luma image which is located on the center of the resultant compact luma image using a mirroring method. Finally, the resultant compact luma image is compressed by a shape-oriented JPEG [39], in which only the meaningful luma values of the resultant compact luma image are compressed. Similarly, the compact subimage $I^{Cb_{3}}$ and the compact subimage $I^{Cr_{2}}$ are compressed using the same codec.

At the client side, the decompressed $Y_{1}Cr_{2}Cb_{3}Y_{4}$ image is converted to the constructed Bayer CFA image $I^{rec,Bayer}$ by the inverse of Equation (1).

### 2.2. The $YD_{g}C_{o}C_{g}$ Method

Extending from the $YC_{o}C_{g}R$ method [40], Malvar and Sullivan [3] proposed an effective $YD_{g}C_{o}C_{g}$ method. Considering each 2 × 2 Bayer CFA block $B^{Bayer}$ in Figure 1a, $B^{Bayer}$ is converted to a 2 × 2 $YD_{g}C_{o}C_{g}$ block where the luma value Y is the average of four Bayer CFA pixel-values in $B^{Bayer}$. The chroma value $D_{g}$ equals the difference between two green pixel-values in $B^{Bayer}$. The chroma value $C_{o}$ equals the difference between the red pixel-value and the blue pixel-value, and the chroma value $C_{g}$ is equal to the difference between the average of two green pixel-values and the average of the red pixel-value and the blue pixel-value. In the $YD_{g}C_{o}C_{g}$ method, the $G_{1}G_{4}R_{2}B_{3}$-to-$YD_{g}C_{o}C_{g}$ transformation is expressed as

(2) $$\left[\begin{array}{c} Y\\ D_{g}\\ C_{o}\\ C_{g} \end{array}\right]=\left[\begin{array}{cccc} \frac{1}{4} & \frac{1}{4} & \frac{1}{4} & \frac{1}{4}\\ -1 & 1 & 0 & 0\\ 0 & 0 & 1 & -1\\ \frac{1}{2} & \frac{1}{2} & -\frac{1}{2} & -\frac{1}{2} \end{array}\right]\left[\begin{array}{c} G_{1}\\ G_{4}\\ R_{2}\\ B_{3} \end{array}\right]$$

The inverse of the above transformation in Equation (2), i.e., the $YD_{g}C_{o}C_{g}$-to-$G_{1}G_{4}R_{2}B_{3}$ transformation, is expressed as

(3) $$\left[\begin{array}{c} G_{1}\\ G_{4}\\ R_{2}\\ B_{3} \end{array}\right]=\left[\begin{array}{cccc} 1 & -\frac{1}{2} & 0 & \frac{1}{2}\\ 1 & \frac{1}{2} & 0 & \frac{1}{2}\\ 1 & 0 & \frac{1}{2} & -\frac{1}{2}\\ 1 & 0 & -\frac{1}{2} & -\frac{1}{2} \end{array}\right]\left[\begin{array}{c} Y\\ D_{g}\\ C_{o}\\ C_{g} \end{array}\right]$$

Because the transformation in Equation (2) and the inverse transformation in Equation (3) only have entries 0, 1, −1, $\frac{1}{2}$, −$\frac{1}{2}$, and $\frac{1}{4}$, according to Equation (3) in [3], only right-shift operations for integer values are needed, leading to a low computational cost benefit. On JPEG-2000, experimental results indicated the compression performance superiority of the $YD_{g}C_{o}C_{g}$ method over the “G channel merging plus color differences” method [10] which outperformed the method by compressing the Bayer CFA image directly.

### 2.3. The YLMN Method

Mohammed et al. [4] proposed a YLMN method for compressing $I^{Bayer}$. Considering a 2 × 2 Bayer CFA Block $B^{Bayer}$, based on the generalized S-transform [41], the lifting-based reversible color transformation [42], and the 2 × 2 unimodular matrix A with

(4) $$A=\left[\begin{array}{cc} \alpha & 1-\alpha \\ 1 & -1 \end{array}\right]$$

for 0 < α < 1, the best value of α is determined as 1/2 and it yields

(5) $$A=\left[\begin{array}{cc} \frac{1}{2} & \frac{1}{2}\\ 1 & -1 \end{array}\right]$$

Based on the differential pulse code modulation (DPCM) and the median edge prediction principle, the $G_{1}R_{2}G_{4}B_{3}$-to-$W_{r}D_{r}W_{b}D_{b}$ transformation is expressed as

(6) $$\begin{array}{cc} \; & \left[\begin{array}{c} W_{r}\\ D_{r} \end{array}\right]=A\left[\begin{array}{c} G_{1}\\ R_{2} \end{array}\right]\\ \; & \left[\begin{array}{c} W_{b}\\ D_{b} \end{array}\right]=A\left[\begin{array}{c} G_{4}\\ B_{3} \end{array}\right] \end{array}$$

To reduce the correlation between $W_{r}$ and $W_{b}$, the $W_{r}D_{r}W_{b}D_{b}$ format in Equation (6) is transformed to the following YLMN format:

(7) $$\begin{array}{cc} \; & \left[\begin{array}{c} Y\\ L \end{array}\right]=A\left[\begin{array}{c} W_{r}\\ W_{b} \end{array}\right]\\ \; & \left[\begin{array}{c} M\\ N \end{array}\right]=\left[\begin{array}{cc} 1 & 0\\ 0 & 1 \end{array}\right]\left[\begin{array}{c} D_{r}\\ D_{b} \end{array}\right] \end{array}$$

Later, Mohammed and Wahid [5] slightly modified the YLMN format in Equation (7) by taking the floor function into account. According to the results proposed by Khan and Wahid [43], Rahman et al. [6] proposed a modified DPCM-based representation for compressing $I^{Bayer}$ in wireless capsule endoscopy applications.

### 2.4. The $Y\Delta C_{b}C_{R}$ Method

Richter and Fößel [8] proposed a $Y\Delta C_{b}C_{r}$ method by modifying the $\tilde{g}dB_{3}R_{2}$ method [7]. After performing the 4 × 4 Haar wavelet transform on the four channels, $\tilde{g}$, *d*, $B_{3}$, and $R_{2}$, the $Y\Delta C_{b}C_{r}$ format is expressed as

(8) $$\begin{array}{cc} \; & Y=\lfloor \frac{R_{2}+2\left\lfloor \frac{G_{1}+G_{4}}{2}\right\rfloor +B_{3}}{4}\rfloor \\ \; & \Delta =G_{1}-G_{4}\\ \; & C_{B}=B_{3}-\left\lfloor \frac{G_{1}+G_{4}}{2}\right\rfloor \\ \; & C_{R}=R_{2}-\left\lfloor \frac{G_{1}+G_{4}}{2}\right\rfloor \end{array}$$

In Equation (8), the *Y* component is the average of the four Bayer CFA pixels of $B^{Bayer}$. Δ denotes the difference between $G_{1}$ and $G_{4}$. $C_{B}$ and $C_{R}$ equal the blue pixel $B_{3}$ and red pixel $R_{2}$ minus the average of $G_{1}$ and $G_{4}$, respectively.

Experimental results indicated that prior to encoding $I^{Bayer}$, as a pre-processing step [8], performing a nonlinear gamma correction on $I^{Bayer}$ can achieve better compression performance when using the $Y\Delta C_{b}C_{r}$ method.

To evaluate the compression performance of the RCT-based compression scheme for $I^{Bayer}$, considering effectiveness, the three RCT-based methods, namely, the $YD_{g}C_{o}C_{g}$ method [3], the YLMN method [4], and the $Y\Delta C_{b}C_{r}$ method [8], are selected as the representatives.

## 3. The Demosaicing-First-Based (DF-Based) Compression Works for Bayer CFA Images

For $I^{Bayer}$, we introduce the related DF-based compression methods, and in particular, the related CSLM (chroma subsampling-then-luma modification) methods are introduced in more detail. We first introduce how to demosaic $I^{Bayer}$ to an RGB full-color image $I^{demo,RGB}$, and then, we introduce how to convert $I^{demo,RGB}$ to a YCbCr image, $I^{YCbCr}$. Finally, three representative methods are selected to evaluate the compression performance of the DF-based compression scheme.

### 3.1. Demosaicing $I^{Bayer}$ to $I^{Demo,Rgb}$ and Then Converting $I^{Demo,Rgb}$ to $I^{Ycbcr}$

In the DF-based compression scheme for encoding $I^{Bayer}$, as depicted at the server side of Figure 3a, $I^{Bayer}$ is first demosaiced to an RGB full-color image $I^{demo,RGB}$.

#### 3.1.1. Demosaicing $I^{Bayer}$ to $I^{demo,RGB}$

To demosaic $I^{Bayer}$ to $I^{demo,RGB}$, a demosaicing method is performed on $I^{Bayer}$ to estimate the other two color channels of each Bayer CFA pixel [44,45,46,47].

Bilinear interpolation [48] is the simplest demosaicing method in which the unknown two-color channels of each Bayer CFA pixel are estimated by averaging its proper adjacent pixels. Kimmel [49] proposed a color difference-based demosaicing method using a template matching approach. Gunturk et al. [50] proposed a demosaicing method using an alternating projection approach. Pei and Tam [51] proposed a demosaicing method using a color correlation approach. Lu and Tan [52] proposed a demosaicing method using the spatial and spectral correlation among the neighboring pixels of each Bayer CFA pixel. Wu and Zhang [53] proposed a demosaicing method using the edge direction information and a soft-decision framework. Lukac and Plataniotis [54] proposed a demosaicing method using normalized color-ratio information.

Hirakawa and Parks [55] proposed an adaptive homogeneity-directed demosaicing method. Chung et al. [56] proposed a demosaicing method using several gradient edge-detection masks and an adaptive heterogeneity-projection technique. Using a generic variational approach, Condat [57] proposed a general demosaicing method for arbitrary CFA patterns. Yang et al. [58] proposed a color difference- and edge sensing-based demosaicing method for arbitrary CFA patterns. Zhang et al. [59] proposed a demosaicing method using local directional interpolation and nonlocal adaptive thresholding.

Kiku et al. [60] proposed a residual interpolation-based demosaicing method. In their method, the missing green values are first estimated by using a bilateral interpolation. Next, a window-based linear relation with two parameters, in which the number of equations is larger than 2, between the estimated green values and the collocated ground-truth red values are constructed. Then, a linear regression technique is applied to solve the two parameters involved in the linear relation. Using the solved two parameters, the missing red values are thus reconstructed. In the same argument, the missing blue values are constructed. To alleviate the spot artifact problem in [60], based on a multiple-window approach, Ye et al. [61] first constructed multiple linear systems, and then the average 2-parameter solution was used to estimate the missing red and blue values, leading to a better smoothing effect. In [62,63], the convolutional neural networks (CNN) based demosaicing methods were proposed. Considering the fact that the green channel has twice the sampling rate and better quality than the red and blue channels in $I^{Bayer}$, Guo et al. proposed a green channel prior-NET-based joint denoising and demosaicing method. Based on a progressive collaborative representation framework, Ni et al. [64] proposed multiple training-and-refining steps to improve the demosaicing performance.

Due to simplicity and effectiveness, as the first step of the DF-based compression method for $I^{Bayer}$, Kiku et al.’s demosaicing method is adopted to demosaic the input Bayer CFA image $I^{Bayer}$ to an RGB full-color image $I^{demo,RGB}$. In the next subsection, the conversion from the demosaiced RGB full-color image $I^{demo,RGB}$ to a YCbCr image $I^{YCbCr}$ is introduced.

#### 3.1.2. Converting $I^{demo,RGB}$ to $I^{YCbCr}$

After demosaicing $I^{Bayer}$ to $I^{demo,RGB}$, $I^{demo,RGB}$ is further transformed to $I^{YCbCr}$ by using the BT.601-5 color conversion [65]:

(9) $$\left[\begin{array}{c} Y_{i}\\ Cb_{i}\\ Cr_{i} \end{array}\right]=\left[\begin{array}{ccc} 0.257 & 0.504 & 0.098\\ -0.148 & -0.291 & 0.439\\ 0.439 & -0.368 & -0.071 \end{array}\right]\left[\begin{array}{c} R_{i}\\ G_{i}\\ B_{i} \end{array}\right]+\left[\begin{array}{c} 16\\ 128\\ 128 \end{array}\right]$$

where for 1≤i≤4, ($R_{i}$, $G_{i}$, $B_{i}$) and ($Y_{i}$,$Cb_{i}$,$Cr_{i}$) denote the triple-values of the *i*th RGB pixel and the *i*th YCbCr pixel in each 2 × 2 RGB block $B^{demo,RGB}$ and the collocated converted 2 × 2 YCbCr block $B^{YCbCr}$, respectively.

Because the human visual system is less sensitive for chroma differences than for luminance, the luma image $I^{Y}$ and the chroma image $I^{CbCr}$ are decorrelated from the converted YCbCr image $I^{YCbCr}$. Therefore, chroma subsampling on each 2 × 2 CbCr block $B^{CbCr}$ is naturally included prior to encoding the YCbCr image [66].

### 3.2. Chroma Subsampling

In this subsection, eight Bayer CFA pattern-independent chroma subsampling methods and five Bayer CFA pattern-dependent chroma subsampling methods are introduced.

#### 3.2.1. The Bayer CFA Pattern-Independent Chroma Subsampling Methods

**Figure 6:**
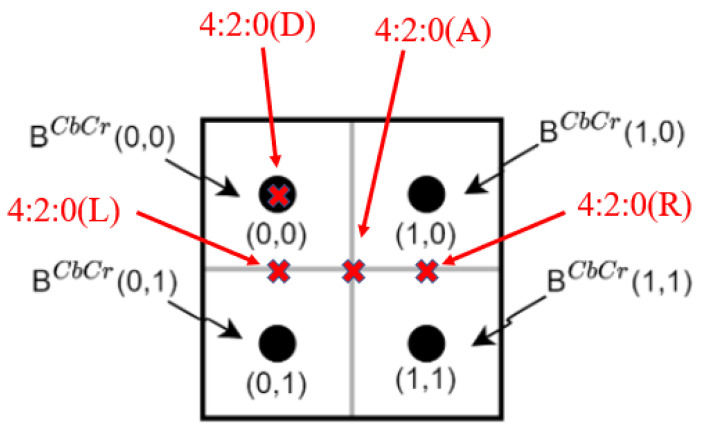
The depiction of 4:2:0(A), 4:2:0(L), 4:2:0(R), and 4:2:0(DIRECT).

For each 2 × 2 chroma block $B^{CbCr}$, 4:2:0(A) averages the four (Cb, Cr)-pair of $B^{CbCr}$ as the subsampled chroma pairs of $B^{CbCr}$. 4:2:0(L) and 4:2:0(R) calculate their chroma pairs by averaging the chroma pairs in the first and second columns of $B^{CbCr}$, respectively. 4:2:0(DIRECT) takes the top-left (Cb, Cr)-pair of $B^{CbCr}$ as the subsampled (Cb, Cr)-pair. Figure 6 depicts the four chroma subsampling methods: 4:2:0(A), 4:2:0(L), 4:2:0(R), and 4:2:0(DIRECT). 4:2:0(MPEG-B) calculates the subsampled (Cb, Cr)-pair of $B^{CbCr}$ by running the 13-tap filter [2, 0, −4, −3, 5, 19, 26, 19, 5, −3, −4, 0, 2]/64 on the top-left position of $B^{CbCr}$.

The Anchor method first performs a 3-tap filter [1, 6, 1]/8 at the leftmost location of each row of $B^{CbCr}$, and then, it performs a 3-tap filter ${\left(\left[0,\;4,\;4\right]/8\right)}^{T}$ at the top-left location of $B^{CbCr}$, where “T” denotes a transpose operator. Based on the new edge-directed interpolation (NEDI) [67], Zhang et al. [25] proposed an IDID chroma subsampling method, and at the client side, NEDI is adopted as the chroma upsampling process. Inspired by the palette mode used for screen content images (SCI) [68], in which each SCI has only a few dominant colors in the background, Wang et al. [26] proposed a JCDU chroma subsampling method, and the bicubic convolution interpolation (BCI) [69] is adopted as the upsampling process at the client side.

However, because the above eight Bayer CFA pattern-independent chroma subsampling methods do not take the Bayer CFA pattern into account, their compression performance is limited. On the other hand, there is room to improve their compression performance.

#### 3.2.2. The Bayer CFA Pattern-Dependent Chroma Subsampling Methods

In this subsection, we introduce the five state-of-the-art Bayer CFA pattern-dependent chroma subsampling methods: the direct mapping (DM) method [27], the COPY-based distortion minimization (CDM) method [28] and the two variants [29,30], and the bilinear interpolation-based distortion minimization (BIDM) method [31].

##### The Direct Mapping (DM) Method [27]

Before presenting the DM method [27], the YCbCr-to-RGB transformation, which is the reverse of the RGB-to-YCbCr transformation in Equation (9), is defined by

(10) $$\left[\begin{array}{c} R_{i}\\ G_{i}\\ B_{i} \end{array}\right]=\left[\begin{array}{ccc} 1.164 & 0 & 1.596\\ 1.164 & -0.391 & -0.813\\ 1.164 & 2.018 & 0 \end{array}\right]\left[\begin{array}{c} Y_{i}-16\\ Cb_{i}-128\\ Cr_{i}-128 \end{array}\right]$$

**Figure 7:**
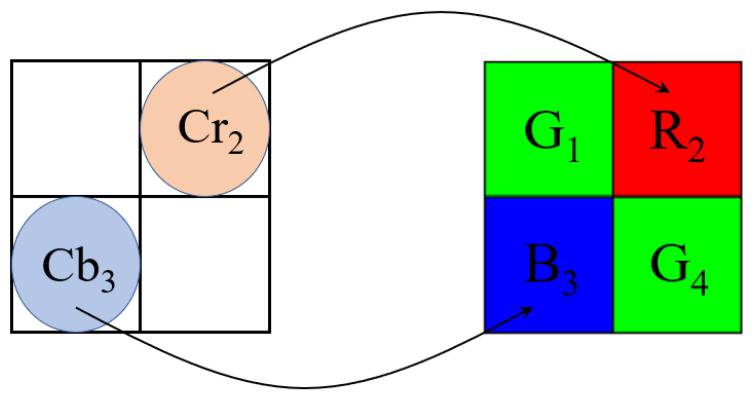
The relation between the subsampled chroma pair ($Cb_{3}$, $Cr_{2}$) and the 2 × 2 Bayer CFA pattern [27].

Chen et al. [27] first observed that the R-color value is dominated by the luma value and the Cb value, and the B-color value is dominated by the luma value and the Cr value. In addition, from the 3 × 3 coefficient matrix in Equation (10), the Cb component has more influence on reconstructing the B pixel than on reconstructing the G pixel. In the same argument, the Cr component has more influence on reconstructing the R pixel than on reconstructing the G pixel. Consequently, the subsampled (Cb, Cr)-pair of $B^{CbCr}$ is set to ($Cb_{3}$, $Cr_{2}$), where the relation between the subsampled chroma pair of $B^{CbCr}$, i.e., ($Cb_{3}$, $Cr_{2}$), and the 2 × 2 Bayer CFA pattern is depicted in Figure 7.

##### The COPY-Based Distortion Minimization (CDM) Method and the Two Variants

Lin et al. [28] first adopted the upsampling process “COPY”, which is called the nearest neighbor (NN) upsampling process supported by some compression standard such as VVC [35], to duplicate the subsampled (Cb, Cr)-parameter of $B^{CbCr}$, denoted by ($Cb_{s}$, $Cr_{s}$), as the four estimated chroma pairs of $B^{CbCr}$ at the server side. Next, they proposed a COPY-based 2 × 2 Bayer CFA block-distortion function to measure the distortion between $B^{CbCr}$ and the 2 × 2 estimated chroma block $B^{est,CbCr}$, and the block-distortion is defined by

(11) $$\begin{array}{cc} D^{Bayer}\left(Cb_{s},Cr_{s}\right) & ={\left(G_{1}-G_{1}^{est}\right)}^{2}+{\left(R_{2}-R_{2}^{est}\right)}^{2}+{\left(B_{3}-B_{3}^{est}\right)}^{2}+{\left(G_{4}-G_{4}^{est}\right)}^{2}\\ \; & =\sum_{i=1}^{4}{\left[\left(1.164Y_{i}+a_{i}Cb_{i}+b_{i}Cr_{i}\right)-\left(1.164Y_{i}+a_{i}Cb_{i}^{est}+b_{i}Cr_{i}^{est}\right)\right]}^{2}\\ \; & =\sum_{i=1}^{4}\left[a_{i}\left(Cb_{i}-Cb_{s}\right)+b_{i}\left(Cr_{i}-Cr_{s}\right)\right){]}^{2} \end{array}$$

with

(12) $$\begin{array}{c} a_{i}=\left\{\begin{array}{cc} 0 & \mathrm{for}\;i\;=\;2\\ -0.391 & \mathrm{for}\;i\;=\;1\;\mathrm{or}\;4\\ 2.018 & \mathrm{for}\;i\;=\;3 \end{array}\right.\\ b_{i}=\left\{\begin{array}{cc} 1.596 & \mathrm{for}\;i\;=\;2\\ -0.813 & \mathrm{for}\;i\;=\;1\;\mathrm{or}\;4\\ 0 & \mathrm{for}\;i\;=\;3 \end{array}\right. \end{array}$$

Applying the differentiation technique to Equation (11), in the real domain, the solution of $\left(Cb_{s},Cr_{s}\right)$ is expressed as

(13) $$\begin{array}{cc} \; & Cb_{s}=\frac{\left(\sum_{i=1}^{4}b_{i}^{2}\right)\left(\sum_{i=1}^{4}a_{i}^{2}Cb_{i}+a_{i}b_{i}Cr_{i}\right)-\left(\sum_{i=1}^{4}a_{i}b_{i}\right)\left(\sum_{i=1}^{4}b_{i}^{2}Cr_{i}+a_{i}b_{i}Cb_{i}\right)}{\left(\sum_{i=1}^{4}a_{i}b_{i}\right){}^{2}-\left(\sum_{i=1}^{4}a_{i}^{2}\right)\left(\sum_{i=1}^{4}b_{i}^{2}\right)}\\ \; & Cr_{s}=\frac{\left(\sum_{i=1}^{4}a_{i}^{2}\right)\left(\sum_{i=1}^{4}b_{i}^{2}Cr_{i}+a_{i}b_{i}-Cb_{i}\right)-\left(\sum_{i=1}^{4}a_{i}b_{i}\right)\left(\sum_{i=1}^{4}a_{i}^{2}Cb_{i}+a_{i}b_{i}Cr_{i}\right)}{\left(\sum_{i=1}^{4}a_{i}b_{i}\right){}^{2}-\left(\sum_{i=1}^{4}a_{i}^{2}\right)\left(\sum_{i=1}^{4}b_{i}^{2}\right)} \end{array}$$

In Lin et al.’s copy-based block-distortion minimization (CDM) method [28], Equation (13) is used to determine the subsampled chroma pair of each 2 × 2 chroma block $B^{CbCr}$; experimental data indicated that the CDM method achieves better compression performance relative to most Bayer CFA pattern-independent chroma subsampling methods. Furthermore, according to the convex function definition in [70], Chung et al. [29] proved that the COPY-based 2 × 2 Bayer CFA block-distortion function in Equation (11) is a convex function because the determinant of the Hessian matrix of $D^{Bayer}\left(Cb_{s},Cr_{s}\right)$ in Equation (11) is equal to 66.1412 (>0). Then, using this convex function property, an iterative CDM (ICDM) method was proposed to obtain a better subsampled (Cb, Cr)-pair of $B^{CbCr}$ when compared with the CDM method [28].

Based on the same differentiation technique used in [28] but considering the 2 × 2 demosaiced RGB full-color block-distortion function, Lin et al. [30] derived that the subsampled chroma pair of $B^{CbCr}$ equals the average chroma pair of the four chroma entries of $B^{CbCr}$. Furthermore, they proposed a “modified 4:2:0(A)” chroma subsampling method that selects the best case among the four average subsampled chroma pairs of $B^{CbCr}$ by considering the four combinations of the ceiling operation-based 4:2:0(A) and the floor operation-based 4:2:0(A). At the client side, the “modified 4:2:0(A)” method adopts the three neighboring (TN) reference pixels-based upsampling process [71]. However, in our experiment, the “modified 4:2:0(A)-COPY” method, where “COPY” denotes the copy interpolation, outperforms the “modified 4:2:0(A)-TN” method. Therefore, the “modified 4:2:0(A)-COPY” method is included in the comparative method instead of the modified 4:2:0(A)-TN method.

##### The Bilinear Interpolation-Based Distortion Minimization (BIDM) Method

**Figure 8:**
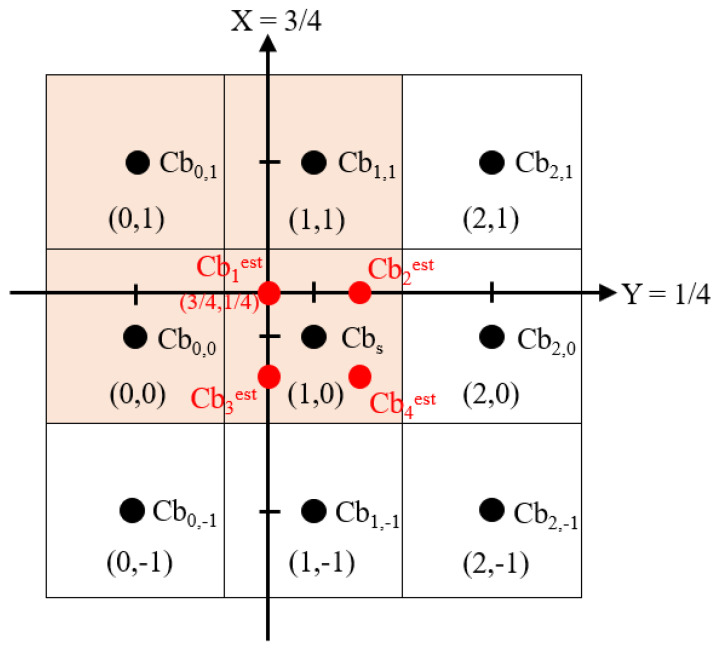
The notations used in the BI-based method for estimating $Cb_{1}^{est}$ at the server side.

To improve the accuracy of COPY-based block-distortion function in Equation (11), Chung et al. [31] proposed a more effective bilinear interpolation-based (BI-based) 2 × 2 Bayer CFA block-distortion function. For simplicity, we just introduce it for each 2 × 2 Cb block $B^{Cb}$. For convenience, let the subsampled Cb parameter of $B^{Cb}$, denoted by $Cb_{s}$, be located at (1, 0) in Figure 8. At the server side, we now describe how to express the estimated top-left entry of $B^{Cb}$, denoted by $Cb_{1}^{est}$, as a function with the parameter $Cb_{s}$. The functions for the other three estimated entries of $B^{Cb}$, namely $Cb_{2}^{est}$, $Cb_{3}^{est}$, and $Cb_{4}^{est}$, can be derived similarly. After estimating the four entries of $B^{CbCr}$, the BI-based 2 × 2 Bayer CFA block-distortion function can be derived.

To estimate $Cb_{1}^{est}$ which is located at (3/4, 1/4), the subsampled chroma parameter $Cb_{s}$ and the three neighboring subsampled Cb values of $B^{Cb}$, namely $Cb_{1,1}$ located at (1, 1), $Cb_{0,1}$ located at (0, 1), and $Cb_{0,0}$ located at (0, 0), are referred to. Because the BI-based distortion minimization (BIDM) method is performed on each 2 × 2 chroma block in a raster scanning order, the three reference subsampled Cb values were obtained in advance. To estimate $Cb^{est,2}$, $Cb^{est,3}$, and $Cb^{est,4}$, some future neighboring reference subsampled Cb values are unknown, but they can be calculated by 4:2:0(A) or the CDM chroma subsampling method in which neither method needs to reference any neighboring subsampled Cb values of the current 2 × 2 Cb block. In our experiment, we adopt 4:2:0(A) to calculate the future subsampled Cb values.

Following the notations in Figure 8 and using the above BI-based approach [31], $Cb_{1}^{est}$ is estimated as

(14) $$Cb_{1}^{est}=\frac{9}{16}Cb_{s}+\frac{1}{16}Cb_{0,1}+\frac{3}{16}Cb_{1,1}+\frac{3}{16}Cb_{0,0}$$

In general, the estimation of $Cb_{i}^{est}$, 1≤i≤4, is expressed as

(15) $$Cb_{i}^{est}=\frac{9}{16}Cb_{s}+\bar{Cb_{i}}$$

with

$$\begin{array}{cc} \; & \bar{Cb_{1}}=\frac{1}{16}Cb_{0,1}+\frac{3}{16}Cb_{1,1}+\frac{3}{16}Cb_{0,0}\\ \; & \bar{Cb_{2}}=\frac{1}{16}Cb_{2,1}+\frac{3}{16}Cb_{1,1}+\frac{3}{16}Cb_{2,0}\\ \; & \bar{Cb_{3}}=\frac{1}{16}Cb_{0,-1}+\frac{3}{16}Cb_{1,-1}+\frac{3}{16}Cb_{0,0}\\ \; & \bar{Cb_{4}}=\frac{1}{16}Cb_{2,-1}+\frac{3}{16}Cb_{1,-1}+\frac{3}{16}Cb_{2,0} \end{array}$$

After estimating the four chroma pairs of $B^{CbCr}$, the estimated 2 × 2 CbCr block $B^{est,CbCr}$, denoted by $B^{est,CbCr}$, the collocated 2 × 2 luma block $B^{Y}$, the Bayer CFA pattern in Figure 1a with $Pat_{1}$ = [$G_{1}$, $R_{2}$, $B_{3}$, $G_{4}$], and Equation (10) are utilized together to reconstruct the estimated 2 × 2 Bayer CFA block $B^{est,Bayer}$ (= [$G_{1}^{est}$, $R_{2}^{est}$, $B_{3}^{est}$, $G_{4}^{est}$]) at the server side. By Equations (9) and (15), the block-distortion $D^{Bayer}\left(Cb_{s},Cr_{s}\right)$ between each 2 × 2 Bayer CFA block and the corresponding 2 × 2 estimated Bayer CFA block is expressed as

(16) $$\begin{array}{cc} D^{Bayer}\left(Cb_{s},Cr_{s}\right) & ={\left(G_{1}-G_{1}^{est}\right)}^{2}+{\left(R_{2}-R_{2}^{est}\right)}^{2}+{\left(B_{3}-B_{3}^{est}\right)}^{2}+{\left(G_{4}-G_{4}^{est}\right)}^{2}\\ \; & =\sum_{i=1}^{4}{\left[\left(1.164Y_{i}+a_{i}Cb_{i}+b_{i}Cr_{i}\right)-\left(1.164Y_{i}+a_{i}Cb_{i}^{est}+b_{i}Cr_{i}^{est}\right)\right]}^{2}\\ \; & =\sum_{i=1}^{4}\left[a_{i}\left(Cb_{i}-\left(\frac{9}{16}Cb_{s}+\bar{Cb_{i}}\right)\right)+b_{i}\left(Cr_{i}-\left(\frac{9}{16}Cr_{s}+\bar{Cr_{i}}\right)\right)\right]{}^{2} \end{array}$$

where $a_{i}$ and $b_{i}$ have been defined in Equation (12).

Furthermore, the determinant of the Hessian matrix of $D^{Bayer}\left(Cb_{s},Cr_{s}\right)$ in Equation (16) equals 6.6216 (>0) [31], and it deduces the convex property of the positive definite block-distortion function $D^{Bayer}\left(Cb_{s},Cr_{s}\right)$ in Equation (16). Using the differentiation technique, it yields that in the real domain, the solution of the subsampled (Cb, Cr)-pair of $B^{CbCr}$, denoted by ($Cb_{s}^{\left(0\right)}$, $Cr_{s}^{\left(0\right)}$), is expressed as

(17) $$\begin{array}{cc} \; & Cb_{s}^{\left(0\right)}=\frac{\left(\sum_{i=1}^{4}b_{i}^{2}\right)\left[\sum_{i=1}^{4}a_{i}^{2}\left(\bar{Cb_{i}}-Cb_{i}\right)+a_{i}b_{i}\left(\bar{Cr_{i}}-Cr_{i}\right)\right]-\left(\sum_{i=1}^{4}a_{i}b_{i}\right)\left[\sum_{i=1}^{4}b_{i}^{2}\left(\bar{Cr_{i}}-Cr_{i}\right)+a_{i}b_{i}\left(\bar{Cb_{i}}-Cb_{i}\right)\right]}{\frac{9}{16}\left[\left(\sum_{i=1}^{4}a_{i}b_{i}\right){}^{2}-\left(\sum_{i=1}^{4}a_{i}^{2}\right)\left(\sum_{i=1}^{4}b_{i}^{2}\right)\right]}\\ \; & Cr_{s}^{\left(0\right)}=\frac{\left(\sum_{i=1}^{4}a_{i}^{2}\right)\left[\sum_{i=1}^{4}b_{i}^{2}\left(\bar{Cr_{i}}-Cr_{i}\right)+a_{i}b_{i}\left(\bar{Cb_{i}}-Cb_{i}\right)\right]-\left(\sum_{i=1}^{4}a_{i}b_{i}\right)\left[\sum_{i=1}^{4}a_{i}^{2}\left(\bar{Cb_{i}}-Cb_{i}\right)+a_{i}b_{i}\left(\bar{Cr_{i}}-Cr_{i}\right)\right]}{\frac{9}{16}\left[\left(\sum_{i=1}^{4}a_{i}b_{i}\right){}^{2}-\left(\sum_{i=1}^{4}a_{i}^{2}\right)\left(\sum_{i=1}^{4}b_{i}^{2}\right)\right]} \end{array}$$

In the integer domain, the subsampled chroma solution in Equation (17) is taken as the initial solution of the iterative BIDM method for obtaining a better subsampled chroma solution of $B^{CbCr}$. In the (k + 1)th iteration of BIDM, if the previous subsampled chroma solution can be replaced by a better solution among the eight neighboring subsampled chroma candidates of ($Cb_{s}^{(k),Bayer}$, $Cr_{s}^{(k),Bayer}$), we set k = k + 1 and repeat the above iterative solution-refinement process; otherwise, we stop the iterative method and report ($Cb_{s}^{(k),Bayer}$, $Cr_{s}^{(k),Bayer}$) as the final subsampled (Cb, Cr)-pair of $B^{CbCr}$. Because the BIDM chroma subsampling method heavily involves the bilinear interpolation, naturally, BIDM selects the bilinear interpolation (BI) as the chroma upsampling process at the client side. Experimental results indicate that the combination BIDM-BI [31] outperforms 4:2:0(A)-BI, CDM-COPY [28], and ICDM-BI [29].

Considering the effectiveness of the above Bayer CFA pattern-dependent chroma subsampling methods, the CDM method, the “modified 4:2:0(A)” method, and the BIDM method are selected as the representatives to evaluate the compression performance of the DF-based compression scheme.

### 3.3. Luma Modification

After introducing the related chroma subsampling works in the CSLM methods, in this subsection, the optimal Bayer CFA pattern-dependent luma modification (OLM) method [33] is first introduced, and then, the difference between the OLM method and Chiu et al.’s non-optimal method [34] is highlighted. For easy exposition, the BIDM-OLM method is used to assist the introduction of the OLM method. After performing the BIDM method on each 2 × 2 chroma block $B^{CbCr}$, the goal of the OLM method is to determine the best modified luma value $Y_{i}^{\prime }$, 1≤i≤4, for the corresponding 2 × 2 luma block $B^{Y}$ such that the 1 × 1 Bayer CFA pixel-distortion can be minimized, achieving better quality of the reconstructed Bayer CFA image.

After performing the iterative BIDM method on the chroma block $B^{CbCr}$, let the subsampled (Cb, Cr)-pair of $B^{CbCr}$ be denoted by ($Cb^{Bayer}$, $Cr^{Bayer}$). Let the two chroma variables $Cb_{i}$ and $Cr_{i}$, 1≤i≤4, in Equation (10) be replaced by $Cb^{Bayer}$ and $Cr^{Bayer}$, respectively. It is intractable to search for a unique modified luma value of $Y_{i}$, 1<=i<=4, which satisfies the three equations in Equation (10) simultaneously. To derive the search interval for determining the best modified luma value $Y_{i}^{\prime }$, 1≤i≤4, for each 2 × 2 luma block $B^{Y}$, we consider $Y_{i}^{R}$, $Y_{i}^{G}$, and $Y_{i}^{B}$, which satisfy

(18) $$\left\{\begin{array}{cc} R_{i}=1.164\left(Y_{i}^{R}-16\right)+1.596\left(Cr^{Bayer}-128\right) & \mathrm{for}\;i\;=\;2\\ G_{i}=1.164\left(Y_{i}^{G}-16\right)-0.391\left(Cb^{Bayer}-128\right)-0.813\left(Cr^{Bayer}-128\right) & \mathrm{for}\;i\;=\;1\;\mathrm{or}\;4\\ B_{i}=1.164\left(Y_{i}^{B}-16\right)+2.018\left(Cb^{Bayer}-128\right) & \mathrm{for}\;i\;=\;3 \end{array}\right.$$

Solving each equation in Equation (18), it yields

(19) $$\left\{\begin{array}{cc} Y_{i}^{R}=\frac{\left(R_{i}-1.596\left(Cr^{Bayer}-128\right)\right)}{1.164}+16 & \mathrm{for}\;i\;=\;2\\ Y_{i}^{G}=\frac{\left(G_{i}+0.391\left(Cb^{Bayer}-128\right)+0.813\left(Cr^{Bayer}-128\right)\right)}{1.164}+16 & \mathrm{for}\;i\;=\;1\;\mathrm{or}\;4\\ Y_{i}^{B}=\frac{\left(B_{i}-2.018\left(Cb^{Bayer}-128\right)\right)}{1.164}+16 & \mathrm{for}\;i\;=\;3 \end{array}\right.$$

By using the contradiction method, it has been proved that the modified luma value $Y_{i}^{\prime }$ can be found in the smaller interval [$Low_{i}$, $High_{i}$] where

(20) $$\begin{array}{c} Low_{i}=\left\{\begin{array}{cc} \left\lfloor Y_{1}^{G}\right\rfloor & \mathrm{for}\;i\;=\;1\\ \left\lfloor Y_{2}^{R}\right\rfloor & \mathrm{for}\;i\;=\;2\\ \left\lfloor Y_{3}^{B}\right\rfloor & \mathrm{for}\;i\;=\;3\\ \left\lfloor Y_{4}^{G}\right\rfloor & \mathrm{for}\;i\;=\;4 \end{array}\right.\\ High_{i}=\left\{\begin{array}{cc} \left\lceil Y_{1}^{G}\right\rceil & \mathrm{for}\;i\;=\;1\\ \left\lceil Y_{2}^{R}\right\rceil & \mathrm{for}\;i\;=\;2\\ \left\lceil Y_{3}^{B}\right\rceil & \mathrm{for}\;i\;=\;3\\ \left\lceil Y_{4}^{G}\right\rceil & \mathrm{for}\;i\;=\;4 \end{array}\right. \end{array}$$

where “⌊·⌋” and “⌈·⌉” denote the floor function and ceiling function, respectively. For 1≤i≤4, it can be verified that the condition “($High_{i}$−$Low_{i}$) = 2” holds, and it thus indicates that the best modified luma value $Y_{i}^{\prime }$ can be determined in constant time such that the pixel-distortion value is minimal, where the pixel-distortion (PD) function is defined by

(21) $$\begin{array}{c} PD\left(Y_{i}\right)=\left\{\begin{array}{cc} {\left(G_{1}-1.164\left(Y_{i}^{G}-16\right)-0.391\left(Cb^{Bayer}-128\right)-0.813\left(Cr^{Bayer}-128\right)\right)}^{2} & \mathrm{for}\;i\;=\;1\\ {\left(R_{2}-1.164\left(Y_{i}^{R}-16\right)+1.596\left(Cr^{Bayer}-128\right)\right)}^{2} & \mathrm{for}\;i\;=\;2\\ {\left(B_{3}-1.164\left(Y_{i}^{B}-16\right)+2.018\left(Cb^{Bayer}-128\right)\right)}^{2} & \mathrm{for}\;i\;=\;3\\ {\left(G_{4}-1.164\left(Y_{i}^{G}-16\right)-0.391\left(Cb^{Bayer}-128\right)-0.813\left(Cr^{Bayer}-128\right)\right)}^{2} & \mathrm{for}\;i\;=\;4 \end{array}\right. \end{array}$$

For $I^{Bayer}$, the previous method proposed by Chiu et al. [34] determined the modified luma value $Y_{i}^{\prime }$ by using the appropriate equality in Equation (19) directly, but it cannot guarantee that the determined modified luma value is the best. Experimental data revealed that the CDM-OLM method [33] can achieve at least 10 dB quality improvement when compared with the pure CDM chroma subsampling method [28].

To evaluate the compression performance of the DF-based compression scheme for $I^{Bayer}$, considering the effectiveness, the three selected representatives are CDM-OLM, “modified 4:2:0(A)”-OLM, and BIDM-OLM.

## 4. Experimental Results

Based on the ground-truth Bayer CFA images collected from the five datasets, namely Kodak, IMAX, SCI, Videos, and CI datasets, the quality and quality–bitrate tradeoff comparison of the reconstructed Bayer CFA and RGB full-color images by using the DF-based compression scheme and the RCT-based compression scheme for encoding Bayer CFA images are demonstrated. In addition, the execution time comparison is also provided.

All considered methods for compressing $I^{Bayer}$ are implemented on a computer with an Intel Core i7-6700 CPU 3.4 GHz and 24 GB RAM. The operating system is the Microsoft Windows 10 64-bit operating system. The program development environment is Visual C++ 2019. The compression standards used to evaluate the compression performance of the considered methods are JPEG-2000 [18,19] and the VVC platform VTM-16.2 [35].

In order to redo the compression experiments for Bayer CFA images, the C++ source codes of the three DF-based compression combinations, namely CDM-OLM-COPY [28,33], “modified 4:2:0(A)”-OLM-COPY [30,33], and BIDM-OLM-BI [31,33], where “COPY” and “BI” denote the upsampling processes used at the client side, can be accessed from the website https://github.com/shuanme/DF-based (accessed on 25 September 2022). The C++ source codes of the three RCT-based compression methods, namely the $YD_{g}C_{o}C_{g}$ method [3], the YLMN method [4], and the $Y\Delta C_{b}C_{r}$ method [8], can be accessed from the website https://github.com/shuanme/RCT-based (accessed on 25 September 2022).

Because it is difficult to access the testing RGB full-color image datasets with real image pipelining parameters, such as the gamma correction coefficients and white balance parameters, at the client side, the reconstructed RGB full-color image is obtained by demosaicing the decompressed reconstructed Bayer CFA image. As mentioned before, the demosaiced Bayer CFA image $I^{demo,Bayer}$ obtained at the server side is used as the ground-truth RGB full-color image for evaluating the quality of the reconstructed RGB full-color image obtained at the client side.

### 4.1. Quality Comparison and Discussion

When setting QP to zero for VTM-16.2 and setting CR to 1 for JPEG-2000, we first compare the quality performance of the reconstructed Bayer CFA and RGB full-color images between the DF-based compression scheme and the CF-based compression scheme for $I^{Bayer}$. Secondly, the discussion of the compression comparison of the two compression schemes is provided.

#### 4.1.1. Quality Comparison and Discussion

Three popular objective quality metrics, namely PSNR, SSIM [36], and FSIM [37], are used to compare the quality performance of the reconstructed Bayer CFA images and the reconstructed RGB full-color images by using all the considered compression methods for Bayer CFA images. PSNR is used to evaluate the average quality of one reconstructed Bayer CFA image, and it is defined by

(22) $$PSNR=\frac{1}{N}\sum_{n=1}^{N}10\log_{10}\frac{255^{2}}{MSE}$$

where denotes the number of the Bayer CFA images in one dataset; MSE (mean square error) equals $\frac{1}{XY}\sum_{i=1}^{X}\sum_{i=1}^{Y}{\left(I^{Bayer}\left(i,j\right)-I^{rec,Bayer}\left(i,j\right)\right)}^{2}$, where $I^{Bayer}$ denotes the ground-truth Bayer CFA image, $I^{rec,Bayer}$ denotes the reconstructed Bayer CFA image, and XY denotes the image size. First, the PSNR value of each dataset is calculated. Next, the average PSNR values of the five considered datasets are calculated as the average PSNR value of one reconstructed Bayer CFA image. By using Kiku et al.’s demosaicing method [60] to demosaic each reconstructed Bayer CFA image $I^{rec,Bayer}$ to a reconstructed RGB full-color image $I^{rec,RGB}$, the CPSNR of $I^{rec,RGB}$ is defined by

(23) $$CPSNR=\frac{1}{N}\sum_{n=1}^{N}10\log_{10}\frac{255^{2}}{CMSE}$$

where CMSE equals $\frac{1}{XY}\sum_{i=1}^{X}\sum_{i=1}^{Y}{\left(I^{demo,RGB}\left(i,j\right)-I^{rec,Bayer}\left(i,j\right)\right)}^{2}$, where $I^{demo,RGB}$ denotes the ground-truth-demosaiced RGB full-color image obtained at the server side.

SSIM is expressed as the product of the luminance mean similarity, the contrast similarity, and the structure similarity between $I^{Bayer}$ and $I^{rec,Bayer}$. To measure the color SSIM (CSSIM) of $I^{rec,RGB}$, the SSIM value of each c-color, c∈{R,G,B}, the image of $I^{rec,RGB}$ is calculated, and then, the average SSIM value of the three calculated SSIM values is used as the CSSIM value of $I^{rec,RGB}$. FSIM utilizes the phase consistency and gradient magnitude to weight the local quality maps for obtaining a feature quality score of $I^{rec,Bayer}$. The color FSIM (CFSIM) value of $I^{rec,RGB}$ is defined as the average FSIM value of the three calculated SSIM values of the three color images of $I^{rec,RGB}$. Interested readers are suggested to refer to the original papers [36,37] for the detailed definitions of SSIM and FSIM, respectively.

Based on the five testing datasets, when setting QP = 0 and CR = 1 for VTM-16.2 and JPEG-2000, respectively, Table 1 tabulates the average PSNR, SSIM, and FSIM performance of the reconstructed Bayer CFA images by using the three representative DF-based compression methods and the three representative RCT-based compression methods, where the individual results on VTM-16.2 and JPEG-2000 are tabulated in different rows. From Table 1, we observe that on VTM-16.2 and JPEG-2000, the $YD_{g}C_{o}C_{g}$ method always achieves the highest PSNR, and the BIDM-OLM method is always ranked second; the BIDM-OLM method achieves the highest SSIM and FSIM, and the $YD_{g}C_{o}C_{g}$ method is ranked second.

The average CPSNR, CSSIM, and CFSIM values of the reconstructed RGB full-color images are tabulated in Table 2. From Table 2, we observe that on VTM-16.2 and JPEG-2000, the $YD_{g}C_{o}C_{g}$ method always achieves the highest CPSNR, and the BIDM-OLM method is ranked second; the BIDM-OLM method always achieves the highest CSSIM and CFSIM, and the $YD_{g}C_{o}C_{g}$ method is always ranked second.

#### 4.1.2. Execution Time Requirement Comparison and Discussion

For each image, the average execution time (in seconds) to transform the input Bayer CFA image to the subsampled YCbCr image for the DF-based compression method or the RCT-based format for the CF-based compression method is tabulated in Table 3. Besides the average execution time of one image for each testing dataset, the average execution time of one image for all five datasets is listed in the last column of Table 3, namely “AVG”. From Table 3, we observe that the dataset “Kodak” takes more time than the other four datasets because of its high resolution. Furthermore, the compression methods in the DF-based compression scheme take more time than the methods in the CF-based compression scheme. Because the average execution time of one image is always less than one second, it can be neglected when compared with the practical encoding time. It is noticeable that each DF-based method (or RCT-based method) can be realized by the usage of the GPU-based parallel computation to reduce the time requirement because each method can be decomposed into many independent subtasks.

### 4.2. Quality–Bitrate Tradeoff Comparison and Discussion

Under different QP and CR intervals, in terms of the BD-PSNR metric, the quality–bitrate tradeoff comparison for all considered compression methods for $I^{Bayer}$ is reported and discussed. To compare the visual effects for the considered compression methods for $I^{Bayer}$, the decompressed Bayer CFA images are further demosaiced by using Kiku et al.’s method to produce the RGB full-color images.

#### 4.2.1. The Quality–Bitrate Tradeoff Comparison

In order to show the average BD-PSNR performance comparison among the considered DF-based compression methods and RCT-based compression methods, we take the method by encoding the testing Bayer CFA images directly as the baseline compression method. According to the reconstructed Bayer CFA images using one considered compression method, under the same bitrate requirement, the quality–bitrate tradeoff metric “BD-PSNR” [38] is used to report the average PSNR gain of the considered compression method over the baseline compression method.

On VTM-16.2, five QP intervals, namely [4, 20], [12, 28], [20, 36], [28, 44], and [36, 51], are used to evaluate the BD-PSNR gains of the reconstructed Bayer CFA images by using the considered methods over the baseline method. On JPEG-2000, five CR intervals, namely [5, 20], [15, 30], [20, 35], [25, 40], and [30, 45], are used to evaluate the BD-PSNR gains of the reconstructed Bayer CFA images by using the considered methods over the baseline method. In Table 4, we observe that on JPEG-2000, the BIDM-OLM method always achieves the best BD-PSNR performance for the five CR intervals. In the same table, on VTM-16.2, the $YD_{g}C_{o}C_{g}$ method achieves the best BD-PSNR gains for the two QP intervals, namely [4, 20] and [12, 28]; the BIDM-OLM method achieves the best BD-PSNR gains for the two QP intervals, namely [20, 36] and [28, 44], and the modified 4:2:0(A)-OLM method achieves the best BD-PSNR gain for the QP interval [36, 51]. It is noticeable that in the QP interval [36, 51], the BD-PSNR gain of the BIDM-OLM method is quite competitive with that of the modified 4:2:0(A)-OLM method, and the difference is only 0.0059 (=3.6112 − 3.6063).

On VTM-16.2, the same five QP intervals are used to evaluate the BD-CPSNR gains of the reconstructed RGB full-color images by using the considered methods over the same baseline method. On JPEG-2000, the same five CR intervals are used to evaluate the BD-CPSNR gains of the reconstructed RGB full-color images by using the considered methods over the baseline method. In Table 5, we observe that the BIDM-OLM method always has the highest BD-CPSNR gains for the five CR intervals on JPEG-2000, and it has the highest BD-CPSNR gains for the three QP intervals, namely [20, 36], [28, 44], and [36, 51], on VTM-16.2. In Table 5, we observe that the $YD_{g}C_{o}C_{g}$ method has the highest BD-CPSNR gains for the two QP intervals, [4, 20] and [12, 28], on VTM-16.2.

In summary, the similar conclusions in Table 4 and Table 5 reveal that on JPEG-2000, the BIDM-OLM method always has the highest BD-PSNR and BD-CPSNR gains for the five CR intervals; on VTM-16.2, the $YD_{g}C_{o}C_{g}$ method has the highest BD-PSNR and BD-CPSNR gains for low and middle QP intervals, and the BIDM-OLM method has the highest BD-PSNR and BD-CPSNR gains for middle and high QP intervals.

#### 4.2.2. The Visual Effect Comparison

**Figure 9:**
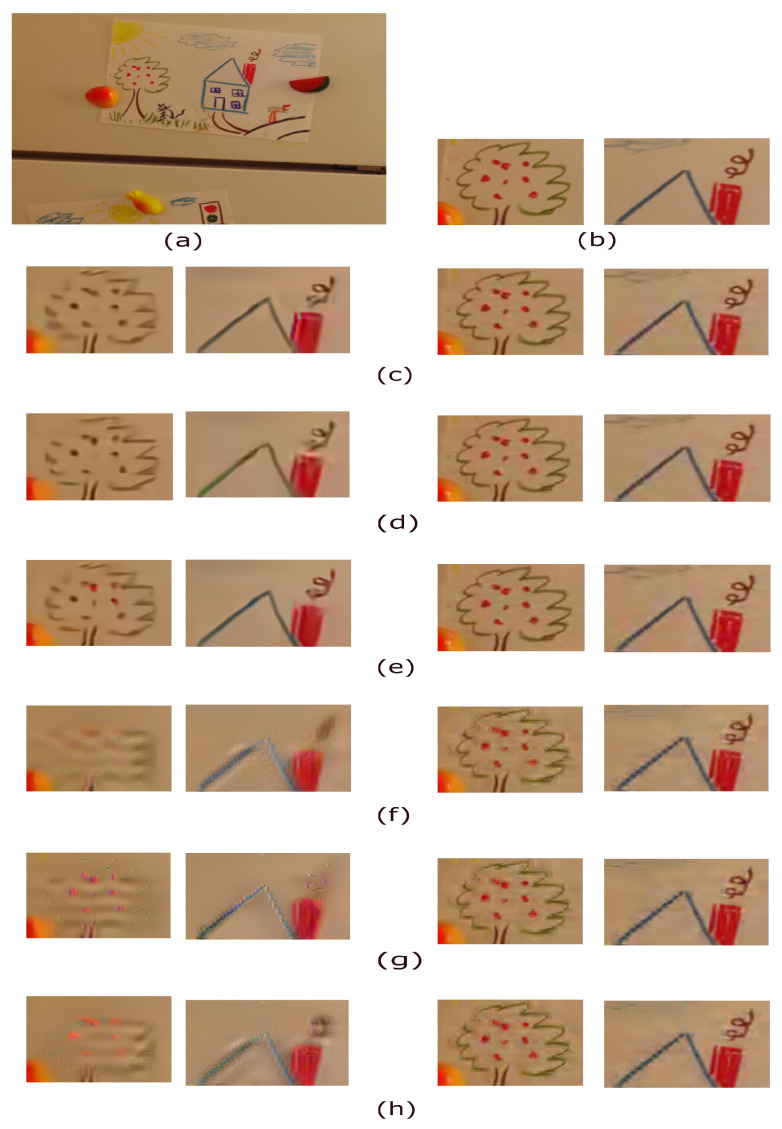
The visual effect comparison for the 13th ground-truth IMAX image, where for each method, the two demosaiced RGB full-color images on the left and on the right are under VTM-16.2 for QP = 44 and JPEG-2000 for CR = 35, respectively. (**a**) The 13th ground-truth IMAX image. (**b**) The amplified subimages. (**c**) CDM-OLM. (**d**) Modified 4:2:0(A)-OLM. (**e**) BIDM-OLM. (**f**) $YD_{g}C_{o}C_{g}$. (**g**) YLMN. (**h**) $Y\Delta C_{b}C_{r}$.

This subsection shows the visual effect comparison among the considered compression methods for $I^{Bayer}$. The testing image example in Figure 9a is taken from the 13th ground-truth IMAX image. After performing the CDM-OLM, modified 4:2:0(A)-OLM, BIDM-OLM, $YD_{g}C_{o}C_{g}$, YLMN, and $Y\Delta C_{b}C_{r}$ methods on the Bayer CFA image of Figure 9b which is cut off from Figure 9a, the demosaiced RGB full-color images are illustrated in Figure 9c–h, respectively, where for each method, the two demosaiced images on the left and on the right are under VTM-16.2 for QP = 44 and under JPEG-2000 for CR = 35, respectively.

Under VTM-16.2, for each method, from the two images on the left in Figure 9c–h, we clearly observe that the DF-based compression methods, particularly the BIDM-OLM method, have better color and texture preservation effects than the RCT-based compression methods. Under JPEG-2000, from the right two images of Figure 9c–h, we observe that the DF-based compression methods still outperform the RCT-based compression methods. The above visual effect observations indicate the visual effect merit of the DF-based methods, particularly the BIDM-OLM method, for one middle and high QP/CR case.

**Figure 10:**
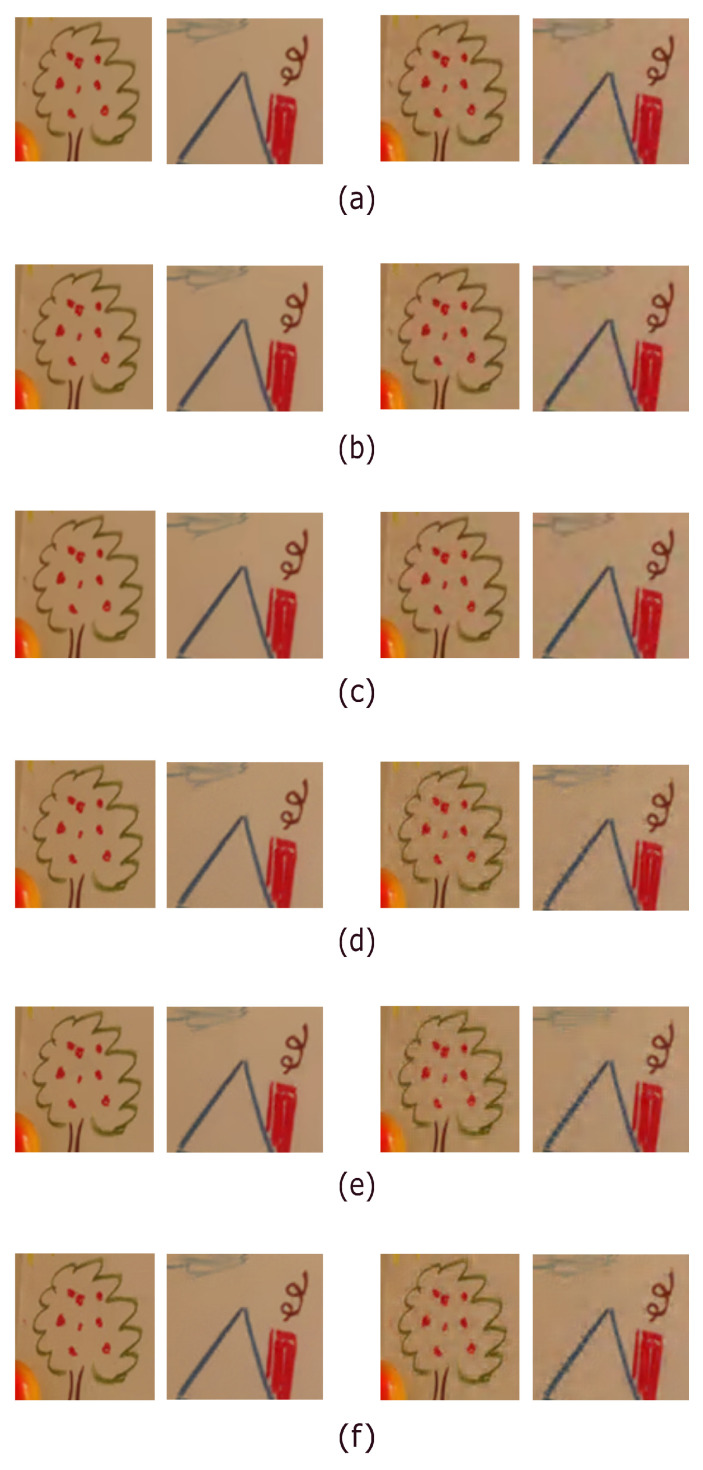
The visual effect comparison for the 13th ground-truth IMAX image, where for each method, the two demosaiced RGB full-color images on the left and on the right are under VTM-16.2 for QP = 24 and JPEG-2000 for CR = 20, respectively. (**a**) CDM-OLM. (**b**) Modified 4:2:0(A)-OLM. (**c**) BIDM-OLM. (**d**) $YD_{g}C_{o}C_{g}$. (**e**) YLMN. (**f**) $Y\Delta C_{b}C_{r}$.

We now consider one low and middle QP/CR case: VTM-16.2 for QP = 24 and JPEG-2000 for CR = 20. After performing the six considered compression methods on the Bayer CFA image of Figure 9b, the demosaiced RGB full-color images are illustrated in Figure 10a–f, where for each method, the two demosaiced RGB full-color images on the left and on the right are under VTM-16.2 for QP = 24 and JPEG-2000 for CR = 20, respectively. For each method, from the two images on the right in Figure 10, under JPEG-2000, we observe that the DF-based compression methods, particularly the BIDM-OLM method, have better color and texture preservation effects in the tree, roof, and chimney parts when compared with the RCT-based compression methods.

Under VTM-16.2 for each method, as shown in the two images on the left in Figure 10, we observe that the DF-based compression methods are quite competitive with the RCT-based compression methods. Note that in the roof part, the BIDM-OLM method has a better visual effect than the $YD_{g}C_{o}C_{g}$ method.

For low QP/CR cases, the visual effect comparison is omitted because the CPSNR values of demosaiced RGB full-color images of the considered compression methods are too high to be visually distinguished. For example, under VTM-16.2 for QP≤20, the CPSNR values are often larger than or equal to 40. Under the two codecs, VTM-16.2 and JPEG-2000 for different QP and CR values, it is suggested that the readers refer to the related experimental results in the above-mentioned two github websites.

## 5. Conclusions and Future Works

We have introduced the compression-first-based compression methods, in particular the reversible color transform-based (RCT-based) compression methods, and the demosaicing-first-based (DF-based) compression methods for Bayer CFA images. Based on five datasets, thorough experiments have been carried out to compare the quality and quality of bitrate tradeoff performance of the RCT-based compression methods and the DF-based compression methods. To the best of our knowledge, this is the first time that such a compression performance comparison has been reported for the two compression approaches for $I^{Bayer}$. Experimental results demonstrated that on JPEG-2000, the BIDM-OLM method always has the highest BD-PSNR and BD-CPSNR gains for different CR intervals. On VTM-16.2, the $YD_{g}C_{o}C_{g}$ method has the highest BD-PSNR and BD-CPSNR gains for low and middle QP intervals, and the BIDM-OLM method has the highest BD-PSNR and BD-CPSNR gains for middle and high QP intervals.

Some future works are addressed below. The first future work is to deploy some image pipelining techniques, such as denoising, gamma correction, and white balancing, into the reconstructed Bayer CFA image at the client side to produce the reconstructed RGB full-color images for the above-mentioned two compression schemes for $I^{Bayer}$. After that, the compression performance is examined. The second future work is to extend the results of this article to RGBW CFA images [72,73,74], which have been widely used in consumer markets and can receive more luminance in the low illumination condition than that of Bayer CFA images [75]. In this future work, the demosaicing method for RGBW images can be adopted from the methods reported in [57,76,77]. The third future work is to take the latest SSIM variants [78,79] into account for enhancing the quality comparison of the considered compression methods for Bayer CFA images.

## Figures and Tables

**Table 1:** When setting QP = 0 and CR = 1 for VTM-16.2 and JPEG-2000, respectively, the quality performance comparison of the reconstructed Bayer CFA images.

Method	Platform	PSNR	SSIM	FSIM
CDM-OLM	VTM-16.2	55.5654	0.99968	0.99988
modified 4:2:0(A)-OLM	VTM-16.2	55.2914	0.99966	0.99987
BIDM-OLM	VTM-16.2	56.8909	0.99977	0.99991
$YD_{g}C_{o}C_{g}$	VTM-16.2	58.0274	0.99972	0.99970
YLMN	VTM-16.2	50.7565	0.99928	0.99964
$Y\Delta C_{b}C_{r}$	VTM-16.2	53.3100	0.99941	0.99966
CDM-OLM	JPEG-2000	55.5956	0.99968	0.99988
modified 4:2:0(A)-OLM	JPEG-2000	55.4404	0.99967	0.99987
BIDM-OLM	JPEG-2000	56.9200	0.99976	0.99991
$YD_{g}C_{o}C_{g}$	JPEG-2000	58.0296	0.99972	0.99990
YLMN	JPEG-2000	50.7568	0.99928	0.99964
$Y\Delta C_{b}C_{r}$	JPEG-2000	53.3106	0.99941	0.99966

**Table 2:** When setting QP = 0 and CR = 1 for VTM-16.2 and JPEG-2000, respectively, the quality performance comparison of the reconstructed RGB full-color images.

Method	Platform	CPSNR	CSSIM	CFSIM
CDM-OLM	VTM-16.2	53.5576	0.99866	0.99983
modified 4:2:0(A)-OLM	VTM-16.2	53.3690	0.99862	0.99983
BIDM-OLM	VTM-16.2	54.4657	0.99892	0.99987
$YD_{g}C_{o}C_{g}$	VTM-16.2	55.2310	0.99888	0.99984
YLMN	VTM-16.2	49.4787	0.99746	0.99959
$Y\Delta C_{b}C_{r}$	VTM-16.2	51.6591	0.99786	0.99959
CDM-OLM	JPEG-2000	53.5863	0.99866	0.99983
modified 4:2:0(A)-OLM	JPEG-2000	53.5154	0.99865	0.99983
BIDM-OLM	JPEG-2000	54.4885	0.99892	0.99987
$YD_{g}C_{o}C_{g}$	JPEG-2000	55.2337	0.99888	0.99984
YLMN	JPEG-2000	49.4793	0.99746	0.99959
$Y\Delta C_{b}C_{r}$	JPEG-2000	51.6602	0.99786	0.99959

**Table 3:** The execution time requirement (in seconds) comparison.

	KODAK	IMAX	SCI	Videos	CI	AVG
CDM-OLM	0.4371	0.0179	0.0307	0.0074	0.0209	0.1028
modified 4:2:0(A)-OLM	2.1914	0.0937	0.1396	0.0381	0.1016	0.5129
BIDM-OLM	0.8362	0.0420	0.0711	0.0151	0.0429	0.2015
$YD_{g}C_{o}C_{g}$	0.0378	0.0017	0.0028	0.0006	0.0018	0.0089
YLMN	0.0364	0.016	0.0028	0.0007	0.0016	0.0086
$Y\Delta C_{b}C_{r}$	0.0547	0.0022	0.0038	0.0010	0.0028	0.0129

**Table 4:** The BD-PSNR gains of the considered methods for $I^{rec,Bayer}$.

Method	Platform	QP [4, 20]	QP [12, 28]	QP [20, 36]	QP [28, 44]	QP [36, 51]
CDM-OLM	VTM-16.2	−3.4486	−0.2175	2.1060	3.4628	3.5398
modified 4:2:0(A)-OLM	VTM-16.2	−3.5834	−0.2550	2.1785	3.5687	3.6122
BIDM-OLM	VTM-16.2	−3.0984	0.1299	2.4742	3.6255	3.6063
$YD_{g}C_{o}C_{g}$	VTM-16.2	0.8212	1.4656	2.1304	2.8782	2.7864
YLMN	VTM-16.2	−1.9614	0.3535	1.5443	2.6012	2.6668
$Y\Delta C_{b}C_{r}$	VTM-16.2	−0.3723	1.2455	2.1641	3.0196	2.8695
		**CR [5, 20]**	**CR [15, 30]**	**CR [20, 35]**	**CR [25, 40]**	**CR [30, 45]**
CDM-OLM	JPEG-2000	1.5667	3.3552	3.7033	3.9106	4.0223
modified 4:2:0(A)-OLM	JPEG-2000	1.5707	3.3740	3.7248	3.9297	4.0396
BIDM-OLM	JPEG-2000	1.6314	3.3979	3.7335	3.9336	4.0401
$YD_{g}C_{o}C_{g}$	JPEG-2000	1.3451	2.7924	3.1319	3.3665	3.5208
YLMN	JPEG-2000	1.1450	2.7286	3.1177	3.3823	3.5621
$Y\Delta C_{b}C_{r}$	JPEG-2000	1.3662	2.9438	3.3218	3.5802	3.7508

**Table 5:** The BD-CPSNR gains of the considered methods for $I^{rec,RGB}$.

Method	Platform	QP [4, 20]	QP [12, 28]	QP [20, 36]	QP [28, 44]	QP [36, 51]
CDM-OLM	VTM-16.2	−2.7093	0.0106	2.2170	3.6186	3.8182
modified 4:2:0(A)-OLM	VTM-16.2	−2.8379	−0.1073	2.1726	3.6543	3.8561
BIDM-OLM	VTM-16.2	−2.4553	0.2697	2.4895	3.7681	3.8773
$YD_{g}C_{o}C_{g}$	VTM-16.2	0.4310	1.0292	1.8324	2.8669	3.0045
YLMN	VTM-16.2	−1.8610	0.0021	1.2083	2.5099	2.8323
$Y\Delta C_{b}C_{r}$	VTM-16.2	−0.4328	0.8685	1.8585	2.9811	3.0826
		**CR [5, 20]**	**CR [15, 30]**	**CR [20, 35]**	**CR [25, 40]**	**CR [30, 45]**
CDM-OLM	JPEG-2000	2.0816	3.8981	4.2366	4.4344	4.5438
modified 4:2:0(A)-OLM	JPEG-2000	2.0211	3.8834	4.2443	4.4536	4.5677
BIDM-OLM	JPEG-2000	2.1005	3.9354	4.2773	4.4794	4.5889
$YD_{g}C_{o}C_{g}$	JPEG-2000	1.8285	3.4338	3.7969	4.0440	4.2071
YLMN	JPEG-2000	1.5357	3.2973	3.7119	3.9886	4.1804
$Y\Delta C_{b}C_{r}$	JPEG-2000	1.8189	3.5772	3.9777	4.2471	4.4271

## Data Availability

Kodak data were obtained from https://www.math.purdue.edu/~lucier/PHOTO_CD/BMP_IMAGES/ (accessed on 10 March 2020), IMAX data were obtained from https://www4.comp.polyu.edu.hk/~cslzhang/CDM_Dataset.htm (accessed on 20 December 2018), SCI data were obtained from ftp://140.118.175.164/SCI (accessed on 26 March 2021), Video data were obtained from ftp://140.118.175.164/CFASS/ (accessed on 25 August 2020), CI data were obtained from https://doi.org/10.1109/TMM.2017.2749162 (accessed on 23 August 2019).
